# Flood dynamics in urbanised landscapes: 100 years of climate and humans’ interaction

**DOI:** 10.1038/srep40527

**Published:** 2017-01-12

**Authors:** G. Sofia, G. Roder, G. Dalla Fontana, P. Tarolli

**Affiliations:** 1 Department Land, Environment, Agriculture and Forestry, University of Padova, Agripolis, viale dell’Università, 16, Legnaro (PD), Italy

## Abstract

Raising interest in the interaction between humans and climate drivers to understand the past and current development of floods in urbanised landscapes is of great importance. This study presents a regional screening of land-use, rainfall regime and flood dynamics in north-eastern Italy, covering the timeframe 1900–2010. This analysis suggests that, statistically, both climate and land-use have been contributing to a significant increase of the contribution of short duration floods to the increase in the number of flooded locations. The analysis also suggests that interaction arises, determining land-use dynamics to couple with climatic changes influencing the flood aggressiveness simultaneously. Given that it is not possible to control the climatic trend, an effective disaster management clearly needs an integrated approach to land planning and supervision. This research shows that land management and planning should include the investigation of the location of the past and future social and economic drivers for development, as well as past and current climatic trends.

The year 2015 was a remarkable year in global policy with the publication of three milestone UN agreements regarding the Disaster Risk Reduction, Sustainable Development Goals and Climate Change^1^. The coincidence of three such treaties is an opportunity of global significance, also to raise interest in the interaction between humans, landscape and climate, to understand the past and current development of disasters in complex landscapes. Urbanized landscapes are one of the most sensitive systems to hydrological extremes, fluctuations and changes^2^,^3^,^4^. Here, hydrogeological disasters such as floods represent one of the most dangerous environmental risks of the current time^5^, with a broad range of impacts on society and the environment. While more evidence is needed to recognise the part played by the climate in this trend^6^, growing human activity in hazard-prone areas has been shown to be a major factor^7^,^8^. Specifically, during history, an enormous, capillary and highly evident system of mechanical devices for channelling and controlling water^9^ shaped urbanised landscapes. Over the centuries numerous changes in land use^10^,^11^,^12^,^13^,^14^,^15^ caused a profound metamorphosis of the natural system^16^. Such alterations have enormous consequences on the flood regime, whose sensitivity to changes tends to increase as the recurrence interval of the rainfall event decreases^10^,^11^,^15^,^17^,^18^. Other influencing factors burdening the responsibility of the high frequency of flood events are changes in people’s wealth, increasing population density and vulnerability^7^, and also, higher rates of urbanisation as a consequence of the economic growth.

While the global process of urbanisation continues, world’s climate changes result in more frequent and more intense flooding^19^. Such increasing trends in flood risk may have severe direct humanitarian and economic impacts and lasting long-term adverse effect in economic growth^20^. Especially river floods in Europe could directly affect more than half a million people a year by 2050 and nearly one million by 2080, as compared to the 200 thousand influenced currently^21^. The predicted steep increase in river flood could mean that related annual damages climb from the current 5.3 billion EUR to up to 40 billion EUR in 2050 and reach 100 billion EUR by 2080, due to the combined effect of climatic change and socio-economic growth^21^. As all these issues are becoming increasingly frequent, the current planning system seems to be inadequate to tackle the landscape vulnerabilities^22^. In this context it is, therefore, timely relevant to analyse impacts and relatively subtle changes in weather and land-use related risks when quantifying present-day flood regimes, to provide essential information to understand the future of flood risk^23^. Achieving this requires a relatively detailed information on topography and asset distribution, as well as information about the climatic settings^21^. In this work, we present a local screening of a long-term analysis to understand the relationship of human land use drivers with the climate dimensions of urban flood dynamics. For the human drivers, we focussed on changes in the built-up areas (or changes in imperviousness, Imp_Ch_) (see Chapter *Methods: Observatory data).* An index of rainfall concentration^24^ –or aggressiveness- (Cl_a_) describes the input given by short but intense rainfall to the total rainfall amount, to investigate the climate dimension. Finally, flood dynamics are studied using an index of flood concentration (Fl_a_) indicating the contribution of short duration floods respect to the increase in the percentage of the flooded locations (see Chapter *Methods: Rainfall and flood concentration*).

The analysis focuses on the Veneto region (north-eastern Italy) (Fig. 1), a paradigmatic example of human-landscape interaction. The region presents a subcontinental climate [average annual temperatures between 10 and 14.4 °C], affecting the whole range of floodplains and the pre-Alpine valleys. The floodplain presents rainfalls equally distributed during the year, with an average yearly value between 600 and 1100 mm, with drier winters and thunderstorms in summer. The pre-Alpine areas present average yearly rainfall of 1100–1600 mm, mostly in autumn and winter. The mountain regions, excluding the pre-Alpine valleys, are located mainly within the cool/cold temperate climate [average annual temperatures between 6 and 9.9 °C for the cool areas, and between 3–5.9 °C for the cold ones], with the highest ranges of the Alps influenced by cold climate [mean annual temperatures below 3 °C]. These zones present average yearly rainfalls of 1600 mm, mostly in late spring and autumn^25^.

Like many other highly developed nations, highly governed landscapes such as the reclaimed areas of Veneto, are landscapes that have been subject to even greater degrees of human manipulation than normal cultural landscapes^26^,^27^. Due to the dispersed character of its urbanisation (diffused city -*Citta diffusa*^28^, Fig. 1a,b), the impact of floods in the region is manifesting with increasingly social effects^29^, especially after rather frequent, short and intense rainfall events (Fig. 1c,d). The proposed analysis applies to any region facing similar issues, for the assessment of flood, climate and land-use interactions at a regional scale.

## Results

### Land use changes

**Figure 2:**
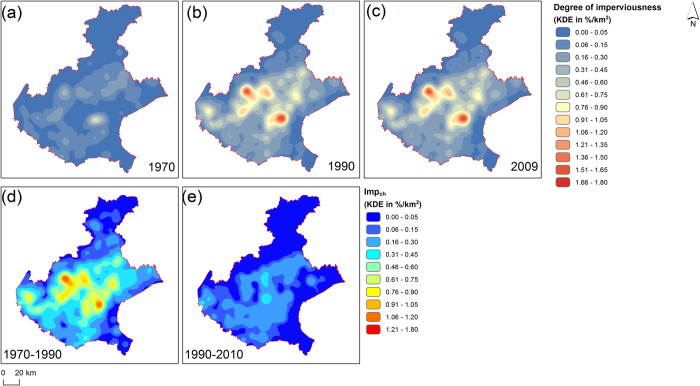
Kernel Density Estimate (KDE) of the degree of imperviousness during the years. (**a**) 1970, (**b**) 1990, (**c**) 2009, and KDE of imperviousness changes (Imp_ch_) between (**d**) 1970–1990 and (**e**) 1990–2009. The map was created with ArcGIS version 10.4 (https://www.arcgis.com/).

Overall the region witnessed a statistically significant trend in the increase of built-up area (*p-value* 0.0275). The imperviousness in the 70s (Fig. 2a) was related to the larger cities in the region (e.g. Padova and Venezia). In 1990 and 2009, the whole region appears to be highly impervious. The highest increase in imperviousness is the one from 1970 to 1990 (Fig. 2d), and it is mostly related to the floodplain area of the region.

**Figure 3:**
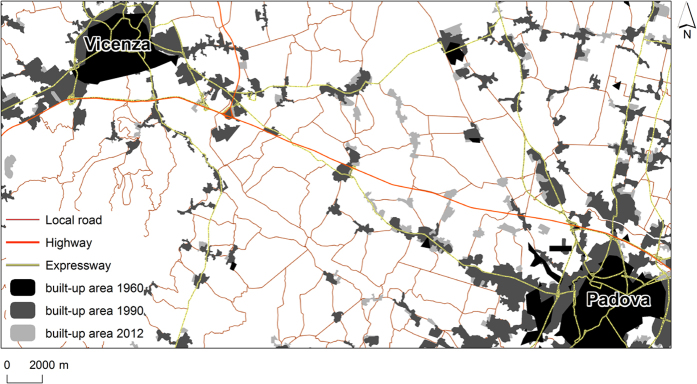
Veneto floodplain. Built-up area in 1970, 1990 and 2012. The map was created with ArcGIS version 10.4 (https://www.arcgis.com/).

The significant changes in imperviousness can be explained by the economic trend of Veneto during the years. In the 1970s, the Gross Domestic Product (GDP) increased more than 30% per year, resulting in Veneto being the second Italian region for GDP^30^. During the 80s, Italy became one of the main economies of Western Europe, thanks to the northern Italy regions. Amongst them, the Veneto economy became an international example. In the 80s the region of Veneto experienced an economic transformation in parallel with a profound change in politics as well^31^. Since 1982, the region’s GDP has kept up a trend of constant growth achieving, from 1993 to 1999, one of the most relevant levels in the whole Europe^30^. In 2000–2007, the GDP grew by 1.3%, showing the greater growing patterns throughout all period analysed except during the crises that penalised the region from 2007 until 2011^30^. The economic growth caused major changes in families’ way of life and local sociability networks. Veneto is characterised by industrial facilities relocation and the diffusion of a manufacturing system based on small firms, whereas small dispersed rural settlements characterise the local social and cultural structure. These interactions resulted, especially in the 1970–1990 timeframe, in dispersed settlements developing within the network of large urban centres^28^,^32^, mostly resulting in loss of agricultural landscape. This high level of urbanisation along with agricultural mechanisation and the regulation of watercourses determined a certain tendency to simplification and unification of the landscape^32^. Differently, the changes over the 1990–2010 period mostly followed transport infrastructures (Fig. 3), and resulted in a low-density suburban development in the periphery of cities, as also witnessed in other countries of Europe^33^.

The analysed urban sprawl and the development of urban land also transformed the properties of soil, reducing its capacity to perform its essential functions. A fully functioning soil for the analysed landscape^34^ can store water for more than 300 m^3^/ha. Covering land with impermeable layers reduces the amount of rain that can be absorbed by the soil. The average changes in imperviousness between 1970 and 1990 (~6%) and between 1990 and 2009 (~2%) can roughly imply in a loss of ~30 M m^3^ and ~10 M m^3^ of water storage over the whole region. In cities with a high proportion of sealed surfaces this loss of storage, especially during heavy rains, can quickly overwhelm drains, causing sewage systems to overflow^35^.

### Climatic trends

**Figure 4:**
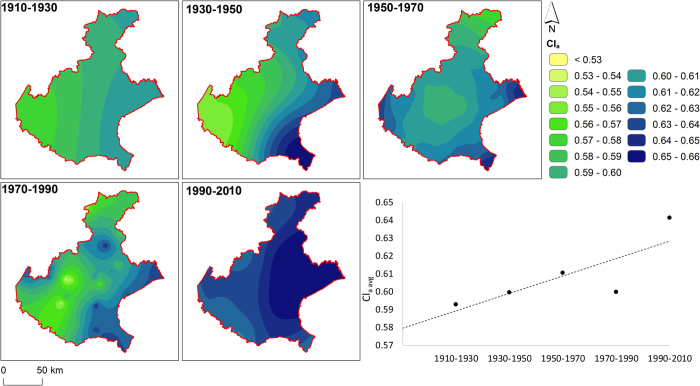
Rainfall concentration (Cl_a_) over the region for the different timeframes and average value derived from these maps. The map was created with ArcGIS version 10.4 (https://www.arcgis.com/).

The climatic trends (Fig. 4) are in line with those already published^36^.

Overall, there is a general statistically significant (*p-value* 0.086) trend of increase of the concentration of the rainfall. The average Climatic aggressiveness (Cl_a_) moves from 0.59 (in 1910–1930) to a value of 0.64. The high value of the last timeframe might be in line with the changes in the decadal climate of the years 1991–2000 and 2001–2010^37^. While Cl_a_ seems to increase constantly during the five timeframes, the timeframe 1970–1990 shows a sensible decrease in the index compared the previous time spans, especially in the central part of the region. Climatically, the region registered a statistically negative trend in the yearly amount of rainfall, more marked in the winter season, with a drastic variation of the winter events in the 80s^36^. These changes are in line with those registered at a wider scale^38^,^39^ and might be correlated with the variability of the global atmospheric circulation^40^.

The coastal area (east/south-east) of the region appears to be the one distinctly different from the rest of the area regarding precipitation concentration, with the higher values of Cl_a_ in each timeframe. This particular climate is due to the proximity to the sea, which causes convective rainfalls to pair with synoptic durations combined to produce exceptionally high rainfall accumulations in this area^39^.

### Flood analysis

**Figure 5:**
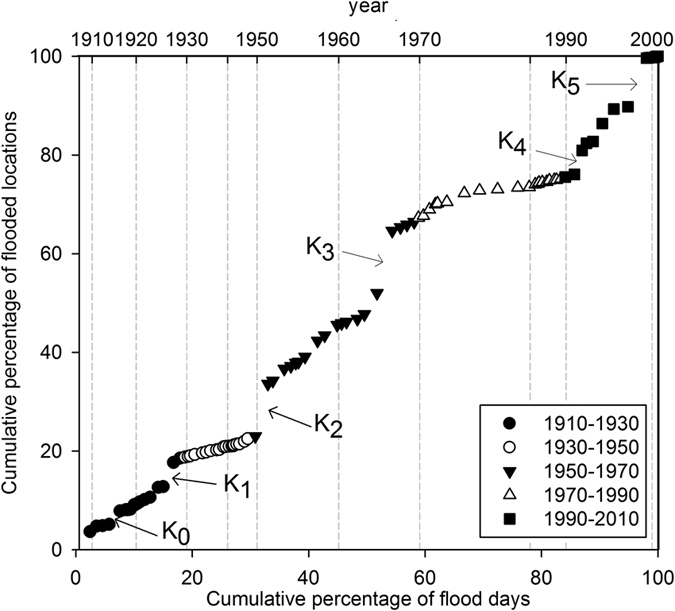
Flood analysis and major past floods in Veneto. Accumulated percentages of flooded sites contributed by the cumulative percentage of flooding days in each year for the whole 1900–2010 timeframe. Knickpoints of the curve are labelled with Ks.

The overall trend between the percentage of flooding days in each year and the number of flooded locations for the whole 1900–2010 timeframe shows the presence of drastic changes in the curve steepness (knick points, labelled with Ks in Fig. 5), implying that during the years fewer days of flood contribute to a notable increase in the percentage of the flooded locations.

**Figure 6:**
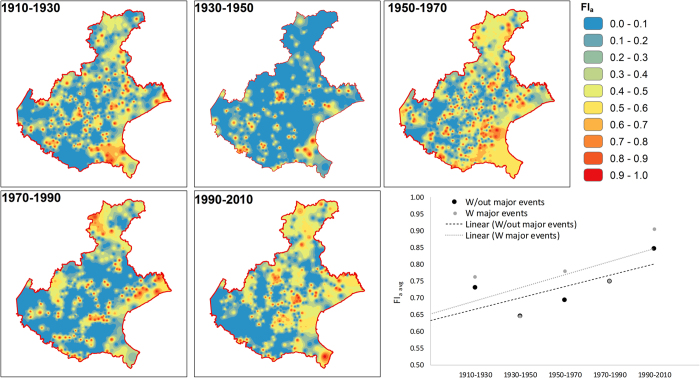
Flood concentration (Fl_a_) over the region and trends during the year, evaluated considering or excluding the major flood events. The map was created with ArcGIS version 10.4 (https://www.arcgis.com/).

The first knickpoint around 1917 (K_0_ in Fig. 5) is probably due to the nature of the considered database that covers only non-systematically the periods 1900 to 1916^41^. The other knickpoints (K_1_ to K_5_) are related to major flood events that affected the region in 1928 (K_1_ in Fig. 6)^41^, 1951 (K_2_ in Fig. 5)^42^, 1966 (K_3_ in Fig. 5)^41^, 1992 (K_4_ in Fig. 5)^41^, 1998 (K_5_ in Fig. 5)^43^.

It is interesting to notice that 1) the analysis of the days of floods VS flooded locations allows to identify clearly major events; 2) between these major events, the trends between days of flood and the percentage of the flooded locations appear to be constant. Analysing the graph considering the timeframes proposed for the research [1910–1930, 1930–1950, 1950–1970, 1970–1990, 1990–2010], it is possible to define statistically significant relationships (*p-value* always < 0.01). In the timeframe 1950–1970, the high slope of the trend is mostly due to the two major events (1951 and 1966). Differently, in the recent decade (1990–2010), the trend seems to be related to a larger number of flood events with a relatively shorter duration (fewer days of floods), which hit a greater number of locations, in addition to the two major floods (1992 and 1998). Thus, suggesting a larger coupling of the land-use and climatic influence for the more recent timeframe.

The average flood concentration (Fig. 6) shows an increasing trend over the considered timeframes, from a value of 0.7 in the period 1910–1930 to a value of 0.9 for the timeframe 1990–2010. Clearly, the major flood events that hit the region have an influence on the index. However the trend appears to be similar, and it becomes more regular when removing such events (1928, 1966, 1992, 1998) from the computation.

The 1910 to 2010 trend is non-significant, both including and excluding the major flood events. The data in the 1910–1930 timeframe are, however, hindered due to the nature of the considered database, that covers only non-systematically the periods 1900 to 1916^41^. Removing the 1910–1930 timeframe makes the trend in Fl_a_ significant (*p-value* 0.089).

### Land-use, Climate and Flood interaction

**Figure 7:**
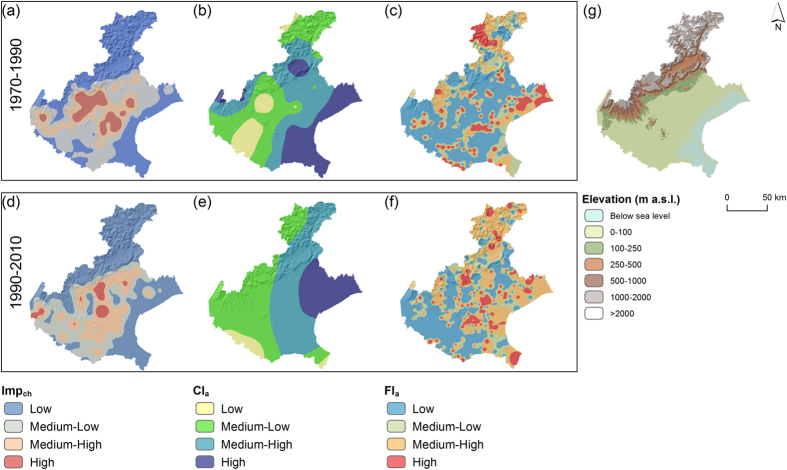
Changes in imperviousness (Imp_ch_ in **a,d**), climatic concentration (Cl_a_ in **b,e**), and flood concentration (Fl_a_ in **c,f**) for the timeframe 1970–1990 (**a**,**b,c**) 1990–2010 (**d**,**e,f**). The values are classified into Low, Medium-Low, Medium-High and High based on a Natural Breaks approach. A map showing the overall elevation of the region is also shown (**g**). The map was created with ArcGIS version 10.4 (https://www.arcgis.com/).

In both 1970–1990 and 1990–2010, the areas having the larger changes in imperviousness (Fig. 7a and d) do not necessarily correspond to regions with the higher climatic concentration (Fig. 7b and e). However, in these areas, the concentration of the floods is high (Fig. 7c and f).

In both timeframes, there is a significant relationship between the flood concentration and the rainfall concentration (Table 1). Individually, Imp_ch_ and Cl_a_ have a significant effect on Fl_a_.

The statistical significance and the changes in order of magnitude (O_m_ changes in Table 1) highlight that despite the overall decrease in the yearly rainfall registered during the years^36^, the increased concentration of the rainfall events (more rain in fewer days) might have resulted in increased concentration of floods, whereas more localities are flooded in fewer days of floods. As well, despite being lower in amount, the changes in imperviousness are still significantly impacting the flood concentration index. In 1990–2010, in addition to the large-scale event of 1998 (K_5_ in Fig. 5), the higher significance was also connected to the influence of climate and urbanisation on local flooding attached to the failure of the urban or peri-urban drainage system^10^,^11^,^15^,^32^.

Furthermore, one should consider that the eastern part of the Region, along the coastline where the climate is more aggressive (e.g. Fig. 7b and e), is characterised by lands lying below sea level (Fig. 7g) that require continuous management of the reclamation networks to stay viable^34^. These areas often witness flooding due to lack of volumes of storage for water within the channels, and the intensity of rainfall events has a significant effect on this^15^,^18^.

**Figure 8:**
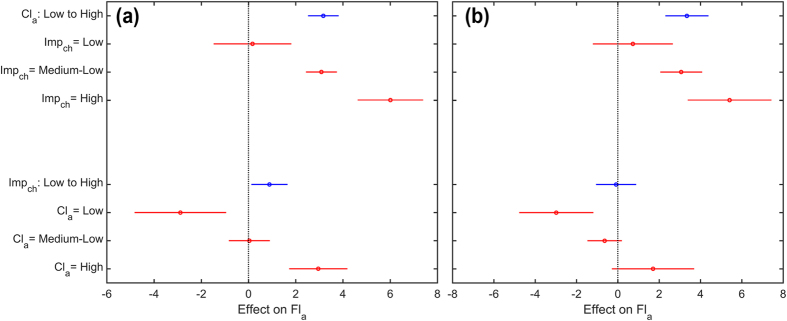
Estimated effects on Fl_a_ of keeping one predictor fixed (Climatic concentration, Cl_a_ in the top half, and changes in imperviousness, Imp_ch_ in the bottom one) while varying the other for the (**a**) 1970–1990 and (**b**) 1990–2010 timeframe. The average effect is shown as a circle, while the horizontal bars are showing the confidence interval for the estimated effect. The blue symbols represent the overall average effect obtained by changing one predictor independently from the other, while the red ones represent the average effect achieved by changing one predictor over different values of the other one.

To better exemplify and analyse the interaction among changes in imperviousness and climatic concentration, Figure 8 shows the estimated effects on Fl_a_ of keeping one predictor fixed (Climatic concentration, Cl_a_ and changes in imperviousness, Imp_ch_) while varying the other. The average effect is shown as a circle, while the horizontal bars are showing the confidence interval for the estimated effect.

**Figure 9:**
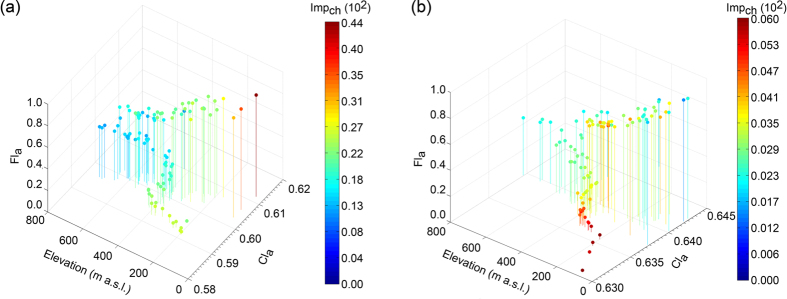
Flood concentration (Fl_a_) for 1970–1990 (**a**) and 1990–2010 (**b**) as related to the landscape topography (elevation m a.s.l.), climatic concentration -Cl_a_ -, and changes in imperviousness -Imp_ch_ -. The percentage changes in imperviousness are shown as multiples of 10^2^. Elevation was computed considering a Digital Elevation Model with a 50 m cell size. Thus they are an approximation of the actual elevation.

For both timeframes, the interaction plots show that the increase in rainfall concentration has a direct (positive) effect on the flood concentration (blue symbol in the top half in Fig. 8a and b). The changes in land use have a greater effect in 1970–1990 respect 1990–2010 (confirming the ANOVA analysis) (blue symbol in the bottom half in Fig. 8a and b): this mostly because the amount of changes in that timeframe is higher (Fig. 7a and b). Overall, the higher the changes in imperviousness, the higher the effect on Fl_a_ at the increase in rainfall concentration (red symbol in the top half in Fig. 8a and b). Changes in imperviousness instead have different implications for the flood concentration, depending on the climatic concentration (red symbols in the bottom half in Fig. 8a and b). For the higher climatic concentration (Cl_a_ = High in Fig. 8a and b), an increase in imperviousness is correlated directly to an increase in the flood concentration (Fl_a_ has a positive variation). However, for Medium-Low values of Cl_a_, changes in imperviousness does not seem to have a great impact on the flood concentration, but still, they imply a slightly positive variation in Fl_a_, at least in the 1970–1990 timeframe. It is interesting to notice, however, that for the least aggressive climate (Cl_a_ = Low in Fig. 8a and b), the increase of imperviousness seems to have a lowering effect on the flood concentration. Different trends in climate and urbanisation depending on the topographic location might explain this latter point (Fig. 9). In both timeframes, part of the region is characterised by an inverse relationship between changes in imperviousness and elevation: urbanisation increases largely in the floodplains, while changes in imperviousness are low (but still positive) for mountain areas. In this same zone, however, the climatic trend is opposite: the rainfall concentration increases with increasing elevation due to the complex role of topography influencing the characteristics of the daily rainfall frequency^44^. The Alpine together with the higher zone of the Pre-alpine territory has an overall low (but increasing at the growth in elevation) level of Cl_a_ that could explain the adverse effects of the increase in imperviousness on the flood concentration in Fig. 8a and b.

A second part of the region, instead, differs in the two timeframes. This area comprises among the rest, the coastal part of the area, where the proximity to the sea produce exceptionally high rainfall accumulations^39^, and urbanisation is highly prominent. In 1970–1990 this part was characterised by an increase in rainfall concentration and a simultaneous growth in imperviousness (Fig. 9a). Especially in the time frame 1970–1990 (Fig. 9a) these contemporary trends result in the highest level of flood concentration. The 1990–2010 period partially differs (Fig. 9b). While the trend for the areas having the lower rainfall concentration is similar to that of 1970–1990 (changes in imperviousness decreases with elevation, while climatic concentration increases), the area with the higher rainfall concentration has a different land-use dynamic. In the 1990–2010 timeframe, there are few areas where urbanisation happens in a relatively larger area of the lower Pre-Alps in addition to the floodplain.

For the rainfall concentration (Cl_up_) and changes in imperviousness (Imp_up_) upstream it is possible to partially draw the same conclusions as described for the local Cl_a_ and Imp_ch_ (Table 1): both parameters, when taken independently, have a significant effect on the flood concentration. However, the significance diminishes in the recent timeframe. The changes in the order of magnitude (O_m_ changes in Table 1) highlight how the changes of imperviousness upstream, despite being still significant, have a lower effect on flood aggressiveness respect to the past (*p-value* increases of 12 orders of magnitude).

The results highlight that in the 1990–2010 timeframe, local changes in imperviousness seem to couple significantly with climate concentration, both considering either the upstream climate alone (Imp_ch_:Cl_up_ in Table 1) or combined with local climate concentration (Cl_a_:Imp_ch_:Cl_up_ in Table 1). For this timeframe, the imperviousness changes -either upstream or local- cannot explain the increase of flood concentration independently from the climate input (Imp_ch_:Imp_up_ in Table 1), while they interacted in the 1970–1990 timeframe with a direct effect on Fl_a_. Despite being significant when taken separately, local climate input and upstream climate do not have a significant interaction in either timeframe. In the 1970–1990 timeframe, upstream climate aggressiveness was more significant when coupling with the upstream land use changes with or without coupling with the local climate changes (Cl_up_:Imp_up_ and Cl_a_:Cl_up_:Imp_up_ in Table 1).

**Figure 10:**
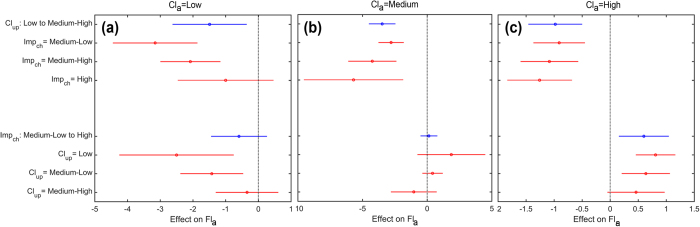
Timeframe 1990–2010. Estimated effects on Fl_a_ of keeping one predictor fixed (Climatic concentration upstream, Cl_up,_ in the top half, and local changes in imperviousness, Imp_ch,_ in the bottom one) while varying the other for (**a**) Low local climate concentration (Cl_a_) (**b**) Average local climate concentration and (**c**) High local climate concentration. The average effect is shown as a circle, while the horizontal bars are showing the confidence interval for the estimated effect. The blue symbols represent the overall average effect obtained by changing one predictor independently from the other, while the red ones represent the average effect achieved by changing one predictor over different values of the other one.

In 1990–2010, when local climate concentration is low (Fig. 10a), the increase of climate concentration upstream (Cl_up_ from Low to Medium-High) has an opposite effect on the local flood concentration. However, when the local imperviousness changes are high (Imp_ch_ = High), the changes in climate upstream lose their effect on Fl_a_ (top half Fig. 10a). An increase of the local imperviousness changes (Imp_ch_ from Medium-Low to High in the bottom half Fig. 10a) has a slightly negative effect on Fl_a_: this effect, however, gets closer to positive when climate concentration upstream is Medium-High.

At the increase of the local climate concentration (Fig. 10b), the effects of land use changes become more evident (Imp_ch_ from Medium-Low to High in the bottom half Fig. 10b) and have the highest effect when the climate upstream is not aggressive (Cl_up_ = Low in Fig. 12b). When local climate is highly concentrated (Fig. 10c), independently from the local land use changes, the increase in climate concentration upstream has an inverse effect on local Fl_a_ (top half Fig. 10c). However, a variation of the local imperviousness changes from Medium-Low to High (bottom half Fig. 10c) has a direct effect increasing the local flood concentration, and this effect is higher for low climatic concentration upstream. This latter point can be explained by the fact that at the local scale climatic concentration has a direct influence on the local flood aggressiveness (Tab. 1). Thus its effect might impact locally, but they might not be transferred downstream.

## Discussion

Keeping in mind that the city is the way in which society is spatially organised to meet the requirements of the financial system^28^, the analysis showed how economic trends and growths during the century could explain the past and current conformation of the impervious area in the region. Concerning the climatic input, despite the negative trend in the overall yearly precipitation^36^, the presented results suggest that this trend has been accompanied by an increase in the concentration of the climate, with short daily events contributing to a larger amount of rainfall. In accordance, numerous studies in literature (with different datasets) showed a similar increase in the mean precipitation intensity for the most recent years, mainly due to a strong positive trend in the contribution of the heavy daily precipitation events^45^,^46^,^47^,^48^. Concerning flood aggressiveness, the observed statistically significant increase implies that in the recent decades, fewer days of flood contribute to a notable increase in the percentage of the flooded locations.

The proposed analysis allows to understand two major aspects that connect land use, climate and flooding: (i) the location of values and key components of the economy provided and provides the primary reason for developments being placed there, but at the same time created and creates risks for the society in terms of flood-exposed goods and thus loss potential (high flood aggressiveness); (ii) in areas where the economy shifts are less evident (e.g. mountain environments, for the analysed region), the changes in rainfall intensity upstream might assume a greater importance, transferring their consequences downstream, depending on the local rainfall intensity.

From the analysis, it can be seen that while climate highly impact the flood trends, but with constant influences, many drivers connected to land use are also prominent, and they change during the years. The economic development and consequent increase in urban areas historically effected the largest extent in the region (in 1970–1990). For the same timeframe, the climatic trend in the area was highly aggressive towards the coastal areas, and population and hence consumption, but also higher exposure, was biased towards the same area. These two changes coupled locally, resulting in the highest flood concentration. Differently, in 1990–2010, the rainfall status showed similar behaviours, but urbanisation increase mostly in a relatively larger area of the lower Pre-Alps in addition to the floodplain, thus interactions between land use and climate might have been transferred downstream, increasing the flood concentration in an area where such issue was already evident in the past. This type of interactions can also be foreseen for the future, although as of now they remain relatively unexplored.

This research emphasises the need for an integrated analysis system that can represent the effects of climate, and the interface with socio-economic effects as both drivers and receptors of flood risk (e.g. land use changes increasing the exposed goods locally, and climate whose effect transfers downstream). Given that the climatic trend cannot be controlled, an effective land management clearly needs an integrated approach for catchment planning and supervision. It should cover the analysis of the location of the past and future drivers for development as well as the past and future drivers of climatic inputs.

## Methods

The analysis focuses on the years 1910 to 2010, excluding 2010. This, to investigate dynamics antecedent to the year of the *All Saints* flood (2010), that caused the largest up to date economic and social impact in the region. This timeframe has been divided four intervals (1910–1930, 1930–1950, 1950–1970, 1970–1990, 1990–2010) considering that 20 years could be long enough for meaningful assessments of climate change^37^, and to capture permanent changes to the built-up system. The availability of information constrained the choice of the time periods for the analyses in terms of time and spatial scale.

### Observatory data

To investigate the changes in floods, a complete database of floods and landslide events is available^41^, with location and dates, that covers systematically the period 1917 to 2000, and non-systematically the periods 1900 to 1916 and 2001 to 2002. The database was updated to the year 2009 by obtaining other records containing information on inundations: bibliographical and reference information, press communications, Veneto Region and the National Civil Protection technical reports, and personal communication with local authorities in charge of the management of rivers. The database reports, aside from the date, the location and the municipality where each event happened.

For the change in land use, the analysis focused on the variations in the built-up areas (or changes in imperviousness). This because changes in artificial coverage have a dual effect on floods: i) a direct hydrologic effect increasing runoff, and ii) an increase in the exposed number of goods. Furthermore, changes in the built-up areas can imply a modification to the drainage system, with changes to the drainage network and conversion to urban drainage system^15^,^32^. The built-up area analysis comprises three reference years (1970, 1990, 2009) and the changes among them. The map of imperviousness for the year 1970 is based on maps produced between 1956 and 1968 by the National Research Council using cadastral datasets at a geographic scale of 1:200,000. The 1990 and the 2009 datasets were derived from the Corine (‘coordination of information on the environment’) Land Cover project (recently taken over by the European Environment Agency, EEA) at a scale of 1:100,000. For these two maps, the analysis focussed on the artificial surfaces excluding those classified as artificial non-agricultural vegetated areas (class 1.4). The three dataset were aggregated at the municipality scale, providing a map of the percentage of imperviousness respect the municipality area. To create a distributed map of the imperviousness for a more quantitative analysis, a kernel-density estimation, hereafter referred to as KDE^49^, was applied. This technique allows, starting from a point location (in this case the centroid of each municipality), to generate a consistent and continuous spatial layer of data^50^, representing the predicted imperviousness or the imperviousness changes throughout the landscape. This solution enabled the use of the different datasets at different resolutions, and it allowed to analyse simultaneously the land use changes analyses with the flood events reported in the flood database and the climatic trends over the region.

For the rainfall dataset, data related to the number of days of rain, and the related cumulated rainfall, were acquired from the automatic weather station network of the Regional Agency for Environmental Protection and Prevention of Veneto (ARPAV), and from the historical values recorded by the network of the Hydrographic Office of the Venice Water (*Ufficio Idrografico del Magistrato alle Acque di Venezia*). Specifically, the products used in this study are rain gauge quantitative precipitation estimates in daily accumulation. The ARPAV dataset comprises rainfall values from 1994 to current days. The Hydrographic Office network, instead, covers the XX century, up to 1994, when the management of the rain-gauge network was delegated to the regional environmental protection agencies.

### Rainfall and Flood concentration

The index of rainfall concentration^24^ –or aggressiveness- (Cl_a_) ranges from 0 to 1, and higher values imply that less days of rain contribute to a notable increase in the accumulated rainfall. The idea of concentration was extended to floods, considering the number of days of flood and the number of flooded sites within the same municipality. Higher values of the flood concentration index (Fl_a_) imply that less days of flood contribute to a notable increase in the percentage of the flooded locations.

**Figure 11:**
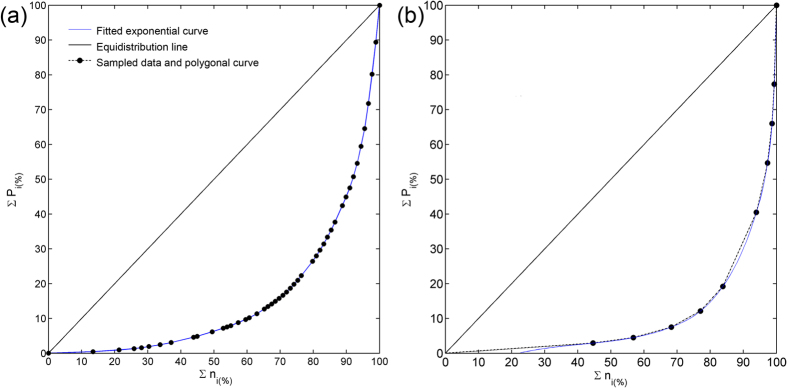
Flood and Climate concentration index. Cumulative percentage ∑n_i(%)_ of rainy days (**a**) and flood days (**b**) plotted against the cumulative percentage ∑P_i(%)._ of rainfall amounts (**a**) or flooded locations (**b**). The fitted exponential curve according to Eq.1 is also shown, as well as the equidistribution line.

For the computation of the indices, the considered variable (rainfall daily cumulative amount or flooded locations within the same municipality) is divided into classes, and it is presented in ascending order. For each class, a midpoint is evaluated, as a sufficiently precise characterization^24^. The number of recorded days (of precipitation for the rainfall characterization, or days of flood for the flood characterization) in each class, or absolute frequency (n_i_), is listed. From these values, the cumulative frequencies are obtained (∑n_i_) by adding the absolute frequencies of all the classes up to the one under consideration, the last one included. n_i_ is multiplied, class by class, with the class midpoint, obtaining the total of each class (P_i_). The cumulative frequencies of Pi (∑P_i_) are computed. Finally, ∑P_i_ and ∑n_i_ are presented in percentages respect to their total. These results give the graphic representation shown in Fig. 11, where the cumulative proportion of rainy days is plotted alongside the cumulative percentage of rainfall amounts (Fig. 11a), and the cumulative percentage of flood days is plotted against the cumulative percentage of flooded locations (Fig. 11b).

In both cases, the resulting polygonal line is markedly exponential and can be represented by an exponential curve of the type


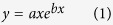


where *a* and *b* can be computed by means of the least-squares method^24^.

The bisector of the graphs in Fig. 11 represents the equidistribution line, an ideal case where the distribution of the variable under consideration is perfect. The area *S* enclosed by the bisector and the polygonal line provides a measure of concentration because the greater the area, the greater the concentration.

Once both the *a* and *b* (Equation 1) constants have been determined, the definite integral of the exponential curve between 0 and 100 is the area *A* under the curve, following Eq. 2


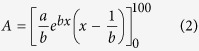


The area *S* compressed by the curve, the equidistribution line and *X* = 100 is the difference between 5000 and the value of Equation (2).

From here, the concentration index is given by


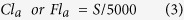


Distributed maps were then obtained by interpolating the local values for each rainfall station (for Cl_a_) or municipality centroid (for Fl_a_) using two different techniques. For the Cl_a_, the ordinary kriging interpolator was chosen to make this study comparable to a previously published research showing values of the Cl_a_ for the region^36^. For the Fl_a_, a simple inverse distance weighting technique was applied: this considering the localised nature of a flood (that is, the farther from the flood break, the lower the damages, generally).

### Downstream effect

To evaluate the effect due to land-use changes and climatic concentration downstream, imperviousness (Imp_up_) and rainfall concentration (Cl_up_) were accumulated downstream, based on the local topography and flow directions^51^. The final accumulated values were standardised according to a min-max normalisation, to have values ranging from 0 to 1 as for the climatic and flood concentration index, where 0, in this case, would imply less climatic concentration or land use changes effects downstream, and 1 the highest contribution. These values should not be taken as absolute values, but rather as an indication of how much changes might have happened upstream, even though there may be no potential for any local impact. In the manuscript, the text will refer to ‘local changes in imperviousness’ or ‘local land use changes’ when speaking about Imp_ch_ and ‘upstream changes in imperviousness’ or ‘upstream land use changes’ when speaking about Imp_up_. Similarly, ‘local climatic concentration’ or ‘local climatic aggressiveness’ will be used for Cl_a_ in opposition to ‘upstream climatic concentration’ or ‘upstream climatic aggressiveness’ for Cl_up_.

### Statistical analyses

The significance of trend of Cl_a_, Fl_a_ and Imp_ch_ during the years was evaluated using a two-tailed Mann–Kendall nonparametric test, with the null hypothesis of a trend absence, against the alternative of a significant trend. The significance threshold level was set^48^ as *p* < 0.10.

The relationship between landuse, climate, and flood aggressiveness relies on the time frames 1970–1990 and 1990–2010, for which there was the availability of all three parameters. The flood concentration was set to be the dependent variable, while imperviousness changes and rainfall concentration were configured to be the explanatory variables. Multiple linear regression models were used to test the ability of each explanatory variable, taken as a single predictor or implying an interaction (the situation in which the simultaneous influence of two variables on a third is not additive) among them, to predict the response variable. To reduce the high variability, the raw flood concentration values were aggregated (binned) into 0.01 intervals, and a mean value for each bin was evaluated. Significant samples for the analysis were therefore obtained by assigning an average value of Fl_a_, say Fl_j_, Cl (Cl_j_) or Imp (Imp_j_) to all locations found having flood concentration in the range of Fl_j_ − 0.01 – Fl_j_ + 0.01. For discussion purposes, the binned values for each parameter have been classified in four classes named High, Medium-High, Medium-Low and Low according to a Natural Breaks Classification, seeking to minimise each class’s average deviation from the class mean, while maximising each class’s deviation from the means of the other groups. The significance of each model was given through an analysis of variance (ANOVA). All the assumptions for the regression models were checked and, in the case of non-linearity between explanatory and response variables, the datasets were opportunely transformed. As for the other trends, the significance threshold level for the ANOVA was set to *p* < 0.10.

## Additional Information

**How to cite this article**: Sofia, G. *et al*. Flood dynamics in urbanised landscapes: 100 years of climate and humans’ interaction. *Sci. Rep.* **7**, 40527; doi: 10.1038/srep40527 (2017).

**Publisher's note:** Springer Nature remains neutral with regard to jurisdictional claims in published maps and institutional affiliations.

## Figures and Tables

**Figure 1:**
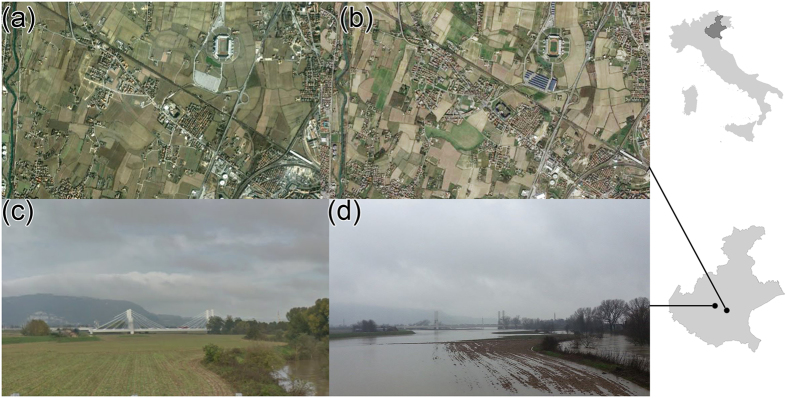
Urban sprawling and examples of a typical flood in the Veneto floodplain. Same area as seen in 2000 [Image© 2016 DigitalGlobe] (**a**) and 2015 [Image© 2016 DigitalGlobe] (**b**). An example of a frequent flood: area as seen in normal condition [Map Data: 2016 Google©] (**c**) and after a few days of rain in 2014 (ph. G.Sofia) (**d**). The map was created with ArcGIS version 10.4 (https://www.arcgis.com/).

**Table 1:** ANOVA analysis showing the significance of effects on Fl_a_ of rainfall concentration (Cl_a_), changes in imperviousness (Imp_ch_), rainfall concentration upstream (Cl_up_), changes in imperviousness upstream (Imp_up_), and their interaction (indicated by the column).

	1970–1990	1990–2010	Δ_p_	O_m_ changes
Rainfall Concentration (Cl_a_)	2.20E-16*	2.20E-16*	+	−2
Change in Imperviousness (Imp_ch_)	3.63E-05*	7.02E-07*	+	−2
Cl_a_: Imp_ch_	2.94E-05*	2.20E-16*	+	−2
Rainfall Concentration upstream (Cl_up_)	2.20E-16*	2.20E-16*	−	0
Change in Imperviousness upstream (Imp_up_)	2.20E-16*	1.59E-03*	−	12
Imp_ch_:Cl_up_	6.50E-03*	6.13E-04*	+	−2
Imp_ch_:Imp_up_	9.46E-11*	ns	−	9
Cl_up_: Imp_up_	2.02E-05*	8.42E-03*	−	2
Cl_a_: Imp_ch_: Cl_up_	5.13E-03*	8.42E-03*	−	0
Cl_a_: Imp_ch_: Imp_up_	1.70E-04*	ns	−	2
Cl_a_: Cl_up_: Imp_up_	ns	5.71E-02*	+	−2

The last two columns show the differences in p-value (Δ_p_) and the changes in the order of magnitude (O_m_ changes) between the two timeframes. For the Δ_p_ column, the negative sign implies a decrease of the significance in the 1990–2010 respect to 1970–1990, positive sign indicates an increase in the statistical significance.

^*^Significant at the 0.10 probability level. ns stands for not significant at the 0.10 probability level.
